# Operator growth from global out-of-time-order correlators

**DOI:** 10.1038/s41467-023-39065-5

**Published:** 2023-06-09

**Authors:** Tianci Zhou, Brian Swingle

**Affiliations:** 1 Kavli Institute for Theoretical Physics, University of California, Santa Barbara, CA 93106 USA; 2 Center for Theoretical Physics, Massachusetts Institute of Technology, Cambridge, MA 02139 USA; 3 Brandeis University, Waltham, MA 02453 USA

**Keywords:** Statistical physics, Quantum information

## Abstract

In chaotic many-body systems, scrambling or the operator growth can be diagnosed by out-of-time-order correlators of local operators. We show that operator growth also has a sharp imprint in out-of-time-order correlators of global operators. In particular, the characteristic spacetime shape of growing local operators can be accessed using global measurements without any local control or readout. Building on an earlier conjectured phase diagram for operator growth in chaotic systems with power-law interactions, we show that existing nuclear spin data for out-of-time-order correlators of global operators are well fit by our theory. We also predict super-polynomial operator growth in dipolar systems in 3d and discuss the potential observation of this physics in future experiments with nuclear spins and ultra-cold polar molecules.

## Abstract (web summary)

Out-of-time-ordered correlators of local operators can quantify information scrambling in quantum many-body systems, but they are not easily accessible in experiments. Here the authors show that their global versions can be used for the same purpose and has been measured in nuclear magnetic resonance experiments.

## Introduction

Out-of-time order correlators (OTOCs) play an important role in the study of quantum chaos. After their introduction years ago^1^, interest in them was recently reignited by the discovery of a quantum generalization of a classical Lyapunov exponent^2–5^. Since then, a large body of work has explored OTOCs in a variety of contexts, in theory^6–11^ and experiment^12–16^. A central question is how to characterize OTOC dynamics in realistic systems, since the Lyapunov behavior seen in large-*N* models is not generic. Here we are particularly interested in OTOCs in systems with power-law interactions^17,18^ motivated by experiments with nuclear spins^19–23^.

**Fig. 1:**
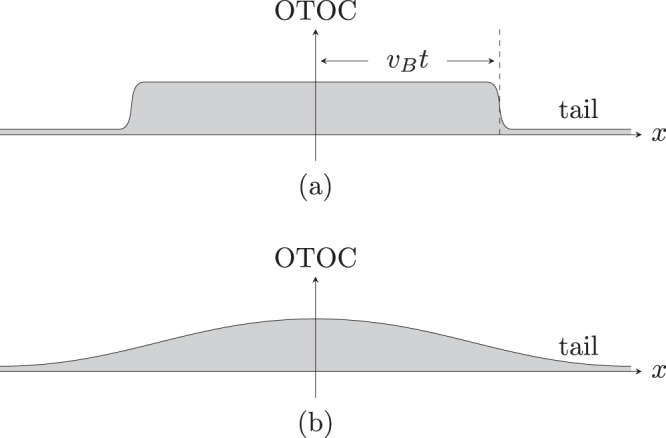
Local OTOC profiles. The local OTOC probes the expansion of the time evolved operator, while the global OTOC is approximately the area under the local OTOC curve. Here *x* denotes the separation between the operators. **a** System with local interactions, where there is a characteristic velocity (the butterfly velocity) *v*_*B*_ for the expansion. **b** System with long-range interactions, where the OTOC curve may have long tails and the light cone may not be sharp.

The microphysics probed by OTOCs is the growth of Heisenberg operators^6,7,9,11,18^. Consider the example of a spin system defined on a spatial grid and let *Z*_*i*_ be a Pauli-*z* operator at site *i* and *Z*_*i*_(*t*) = *e*^*iHt*^*Z*_*i*_*e*^−*iHt*^ be the corresponding Heisenberg operator. The infinite temperature OTOC between *Z*_*i*_ and the Pauli-*x* operator at site *j* is ${{{{{{{\rm{tr}}}}}}}}([{Z}_{i}(t),\, {X}_{j}]{[{Z}_{i}(t),\, {X}_{j}]}^{{{{\dagger}}} })/{{{{{{{\rm{tr}}}}}}}}({\mathbb{I}})$. At time zero, the OTOC is a delta function in space, since *Z*_*i*_ and *X*_*j*_ commute unless *i* = *j*. At later times, the operator *Z*_*i*_(*t*) grows in complexity and spreads in space leading the OTOC to become non-zero when *j* is within a ball of time-dependent radius centered at *i*. This behavior is illustrated in the top panel of Fig. 1.

There is now intense interest in validating and extending this basic physical picture in well-controlled experimental systems. For example, drawing on the tight connections between operator growth and holographic models of quantum gravity^2–5^, experiments probing OTOCs might point to the way to new models with holographic duals. Such experiments may also help us understand what replaces simple Lyapunov-like operator growth in more realistic systems.

However, experiments in this area are typically quite challenging, as they require either time evolution with both *H* (forward) and −*H* (backward)^24,25^ or a large number of randomized measurements^26^ or precision measurements of a small purity-like signal^25^. Local control and readout are also often required depending on the precise setup.

Remarkably, there is a class of experimental systems in which backward time evolution is approximately possible: magnetic resonance experiments with nuclear spins^27–29^. The nuclear magnetic resonance (NMR) community has been measuring relatives of many-body OTOCs since at least the 1970s with the development of the magic echo technique^27,28,30^. However, there is a major complication: one typically has no local control or readout in these experiments, so what one gains access to are OTOCs of global operators. In the spin example, this means it is possible to measure OTOCs between global spin operators, such as ${{{{{{{\rm{tr}}}}}}}}([Z(t),\, X]{[Z(t),\, X]}^{{{{\dagger}}} })$, where *Z* = ∑_*i*_*Z*_*i*_ and *X* = ∑_*i*_*X*_*i*_. It is not clear a priori how these global measurements relate to the local OTOCs that are more commonly studied.

Motivated by these observations, we argue that the key physical property probed by local OTOCs, namely the size of growing operators, is also diagnosed by global OTOCs^31,32^. Under a few conditions which we expect are generic to chaotic evolutions, we show that the global OTOC is proportional to the “area under the local OTOC”, i.e., the gray region in Fig. 1.

**Fig. 2:**
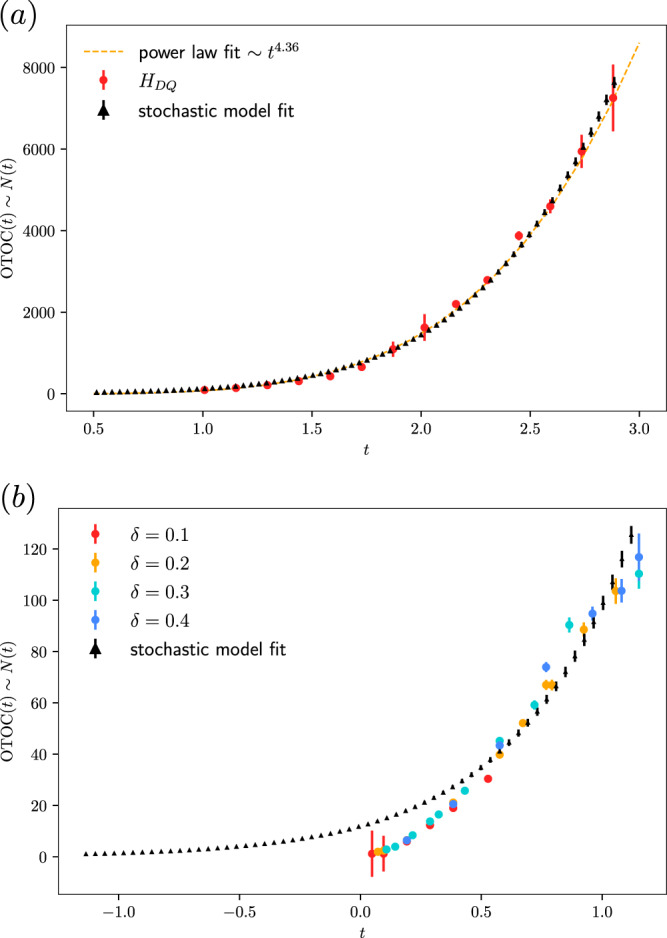
Fits with adamantane data. A two-parameter fitting of our stochastic model results (solid triangle) with the adamantane measurements (circle) of the global out-of-time ordered commutators. Error bar denotes the standard error of the mean in either the mean value from simulation (over 10^3^ samples) or experiments. Experimental data is displayed with the permission of the authors. The unit of time here is 0.4 ms. **a** Data for double quantum Hamiltonian *H*_*DQ*_ evolution taken from Fig. 10 of ref. ^50^. The cluster size reaches almost 10^4^ with a power law fit [yellow dash] of a mysterious exponent 4.36. The stochastic model has a time shift of −0.62 and *K* ≈ 1.77. **b** Data for dipolar Hamiltonian *H*_*YY*_ evolution taken from Fig. 3 of ref. ^23^ for different scaling parameter *δ* (from 0.1 (red) to 0.4 (blue)). The stochastic model has a time shift of −0.79 and *K* ≈ 2.27. Source data are provided as a Source Data file.

In light of this result, we revisit multiple quantum coherence measurements on the nuclear spins in adamantane with cluster sizes of up to 10^4^^22^. Based on the structure of this material, we propose a simplified stochastic model which can be analyzed via Monte Carlo sampling and find that a two-parameter fit of the model prediction agrees well with experimental data (Fig. 2). Our results support the hypothesis that quantum information scrambling with up to ~10^4^ spins has been observed in adamantane, and we predict new phenomena including super-polynomial scrambling at longer times. Finally, we discuss the extension to similar experiments with ultra-cold polar molecules^13,33,34^, finding that similar operator growth can be observed with modest gains in density and coherence time.

## Results

### Global OTOC as the area under the local OTOC

As outlined above, in some quantum simulation platforms, it is possible to measure a global version of the OTOC. We consider the case of spin-1/2 degrees of freedom arising from nuclear spins. Let *X*_*a*_, *Y*_*a*_, *Z*_*a*_ be the Pauli matrices for spin *a*. We consider infinite temperature global OTOCs built from commutators of the total spin, for example, we can take the commutator of the total *z* spin *Z* = ∑_*a*_*Z*_*a*_ and its time evolved form, *Z*(*t*) = *e*^*iHt*^*Ze*^−*iHt*^,

1 $${C}_{g}(t)=-\frac{{{{{{{{\rm{tr}}}}}}}}({[Z(t),\, Z]}^{2})}{{{{{{{{\rm{tr}}}}}}}}(ZZ)}.$$

The normalization factor ${{{{{{{\rm{tr}}}}}}}}(ZZ)$ is equal to ${\sum }_{a}{{{{{{{\rm{tr}}}}}}}}({Z}_{a}Z)=N{2}^{N}$, where *N* is the total number of spins. To relate to the linear size *L*, a system in *d* dimensions has *N* ~ *L*^*d*^.

By contrast, the quantum chaos literature primarily studies local OTOCs, which only involve commutators of local spins. One example is

2 $${C}_{ab}(t)=-\frac{{{{{{{{\rm{tr}}}}}}}}\left({[{Z}_{a}(t),\, {Z}_{b}]}^{2}\right)}{{2}^{N}},$$

which depends on two spin labels *a* and *b*. The operator at *a* initially commute with *Z*_*b*_. Time evolution expands the support of *Z*_*a*_ away from *a*, so that it no longer commutes with *Z*_*b*_ at *b*. Thus the local OTOC probes the expansion of the time evolved operator *Z*_*a*_(*t*).

To relate the global and local OTOCs, we expand the global spins in Eq. (1). The numerator becomes a four-fold summation ${\sum }_{abcd}{{{{{{{\rm{tr}}}}}}}}([{Z}_{a}(t),\, {Z}_{b}][{Z}_{c}(t),\, {Z}_{d}])$. Identifying the *C*_*ab*_(*t*) as the “diagonal” contribution when *a* = *c* and *b* = *d*, *C*_*g*_ has the following decomposition in terms of the diagonal and off-diagonal parts,

3 $${C}_{g}=\frac{1}{N}\mathop{\sum}\limits_{ab}{C}_{ab}-\mathop{\sum}\limits_{\begin{array}{c}a\ne c\\ {{{{{{{\rm{or}}}}}}}} \, b\ne d\end{array}}\frac{{{{{{{{\rm{tr}}}}}}}}([{Z}_{a}(t),\, {Z}_{b}][{Z}_{c}(t),\, {Z}_{d}])}{N{2}^{N}}.$$

In a quantum chaotic system, we argue that the off-diagonal term is negligible compared to the diagonal term at long times. To illustrate the reasoning, consider the case when *a* = *c*, *b* ≠ *d*. The off-diagonal OTOC can be rewritten as ${{{{{{{\rm{tr}}}}}}}}([[{Z}_{a}(t)/{2}^{N/2},\, {Z}_{b}],\, {Z}_{d}]{Z}_{a}(t)/{2}^{N/2})$. The operator *Z*_*a*_(*t*)/2^*N*/2^ is normalized with respect to the operator inner product $(A,\, B)={{{{{{{\rm{tr}}}}}}}}({A}^{{{{\dagger}}} }B)$. Equivalently, its coefficients in terms of the Pauli string basis can be viewed as an amplitude of a wavefunction. After taking a double commutator, the amplitudes are (completely) exchanged. Assuming the amplitudes are random, which is reasonable after a chaotic evolution, they will destructively interfere. But if *b* = *d*, amplitudes with *X* or *Y* at *b* are not exchanged. They are in phase, and thus can produce a much larger contribution to *C*_*g*_. This argument can be quantitatively carried out, see Methods. We also refined the reasoning to the case with interacting quantum circuits (see Supplementary Section III). Numerical computations in small systems confirm the observation (See Methods) that the sum of the off-diagonal terms is indeed negligible.

The global OTOC, after neglecting the off-diagonal terms, is approximately $\frac{1}{N}{\sum }_{ab}{C}_{ab}$, or ∑_*b*_*C*_*ab*_ for any *a* when inhomogeneities such as edge effects can be ignored. The global OTOC is thus the sum of local OTOCs ∑_*b*_*C*_*ab*_. It is an integral of the local OTOC, which is the “area” (literally, in 1d) under the local OTOC curve.

In a locally interacting system, there is a typical velocity *v*_*B*_ that characterizes the spreading of *Z*_*a*_(*t*). The local OTOC is almost 0 when ∣**x**_*b*_ − **x**_*a*_∣ ≫ *v*_*B*_*t*, and approaches an order unity value in a chaotic system when ∣**x**_*b*_ − **x**_*a*_∣ ≪ *v*_*B*_*t*, see Fig. 1a. Assuming homogeneity, we have ${C}_{g}(t) \sim {({v}_{B}t)}^{d}$. In long-range interacting systems, the light cone cutoff may no longer be sharp, see Fig. 1b; but the interpretation as the area under the local OTOC curve still applies.

### Global OTOC in nuclear magnetic resonance experiments

We now test our theory for global OTOCs using existing NMR data. The relevant experiments use nuclear spins to form interacting quantum magnets. In the rotation frame of the strong magnetic field in *z* direction, the nuclear spins are evolved by the dipolar interaction which decays as distance cubed. External radio frequency waves can manipulate the global spin variable. Considerable effort is devoted to design the pulse sequences that, when combined with time evolution under the dipolar interaction, interesting effective Hamiltonians can be generated.

For example, in the adamantane experiments we discuss below, researchers can engineer a double quantum Hamiltonian,

4 $$\begin{array}{r}{H}_{{{{{{{{\rm{DQ}}}}}}}}}=\mathop{\sum}\limits_{a\ne b}{D}_{ab}({X}_{a}{X}_{b}-{Y}_{a}{Y}_{b}),\end{array}$$

where each term changes the total *z* spin by ± 2. The interaction coefficient is

5 $${D}_{ab}=J\frac{(3{\cos }^{2}{\theta }_{ab}-1)}{2{r}_{ab}^{3}},$$

where *r*_*ab*_ is the distance between the two spins, *r*_*ab*_ = ∣**r**_*a*_ − **r**_*b*_∣, and *θ*_*ab*_ is the angle between **r**_*a*_ − **r**_*b*_ and the external magnetic field (*z* direction). A “Y” convention dipolar Hamiltonian can also be engineered,

6 $${H}_{{{{{{{{\rm{Y\; Y}}}}}}}}}=\mathop{\sum}\limits_{a\ne b}{D}_{ab}({Y}_{a}{Y}_{b}-{X}_{a}{X}_{b}-{Z}_{a}{Z}_{b}).$$

Moreover, for each of these Hamiltonians, one can design pulse sequences that correspond to evolving with both *H* and − *H*. As reviewed in Supplementary Section I, this enables measurement of the global OTOC,

7 $${C}_{g}=-{{{{{{{\rm{tr}}}}}}}}\left({[{e}^{-iHt}Z{e}^{iHt},\, Z]}^{2}\right)/\left(N{2}^{N}\right).$$

The ability to evolve forward and backward with these Hamiltonians is approximate, but we focus on the ideal situation.

We choose the material adamantane as an example because of the availability of large scale global OTOC data. Adamantane is a solid polycrystal at room temperature. The crystal structure is face-centered cubic (fcc) with one adamantane molecule (*C*_10_*H*_16_) at each lattice site. The Hydrogen protons comprise the active nuclear spins, so there are 16 spin-1/2s per lattice site. Adamantane also has the peculiar feature that the molecules tumble in place in the lattice at relevant temperatures due to their nearly spherical nature.

The measurement of global OTOCs in adamantane molecules dates back to the 1980s under the name of multiple quantum coherences^27,28,35–38^ (MQC), although at that time only a handful of coherent spins were involved^39^. More recently, thanks to improved coherence times^40^ and the scaled Hamiltonian technique^41^, the number of coherent spins can be as large as 10^4^^22^.

Previous works had developed stochastic model in the space of coherent spin cluster size (*K*) and level of multiple quantum coherence (*n*) to understand the dynamics of MQC. This approach can fit the data when *K* is small. But it ignores the spatial structure of the interaction and predicts an exponential growth of the global OTOC in time, see Supplementary Section II. Other non-stochastic models, such as the Levy-Gleason model^42,43^, encounter the same issue. As experiments push to larger sizes and longer times, the spatial structure of the interactions becomes important.

We propose a model that better accounts for the spatial structure of the dipolar interactions. In adamantane, the dipolar interaction exists between any pair of proton nuclear spins. Its strength has an angular dependence $3{\cos }^{2}{\theta }_{ab}-1$ (Eq. (6)), where *θ*_*ab*_ is the angle between the *z* axis and vector **r**_*ab*_ from *a* to *b*. Unusually, the adamantane molecules constantly tumble in place, with a time scale much shorter than that of the intra-molecular dipolar interaction. Hence if *a*, *b* are in the same molecule, the fast tumbling means that the vector **r**_*ab*_ has an ergodic trajectory over the sphere and the coupling will average to zero. For a similar reason, fast tumbling implies that all nearest-neighbor inter-molecular couplings time-average to the same value and decay as $\frac{1}{{r}^{3}}$.

We propose a further simplification that is expected to capture the leading time-dependence of operator growth. We consider a model where the interactions between *i* and *j* include all possible terms,

8 $$H=\mathop{\sum}\limits_{ij}{K}_{ij}\mathop{\sum }\limits_{a,b=1}^{16}\mathop{\sum }\limits_{\mu,\nu=0}^{3}{({B}_{\mu \nu }(t))}_{ij}^{ab}{({\sigma }^{\mu })}_{i}^{a}{({\sigma }^{\nu })}_{j}^{b},$$

where ${({B}_{\mu \nu }(t))}_{ij}^{ab}$ are independent Gaussian white noise variables. This modification destroys most of the symmetries, but retains the essential structure of the long-range interaction and a large number of spins (in this case, *M* = 16) on each site. The assumption here is that the dipole interaction in three dimensions is non-integrable and the completely chaotic Brownian Hamiltonian in Eq. (8) can capture the dynamics at long times. There are numerical evidences to support non-integrability in similar two dimensional models^44^, although in one dimension experiments show that dipolar Hamiltonians could well be close to integrable^19^. *K*_*ij*_ is proportional to $\frac{1}{|{{{{{{{{\bf{r}}}}}}}}}_{i}-{{{{{{{{\bf{r}}}}}}}}}_{j}{|}^{3}}$. We notice that the conserved charges (total energy, and total *y* spin in Eq. (6)) are orthogonal to the operator ∑_*a*_*Z*_*a*_. Moreover, the coherent evolution of the conserved parts is neglegible in both the diagonal and non-diagoal OTOCs, and therefore can be ignored.

In our previous works, we analyzed the asymptotic light cone structure of local OTOCs in models like Eq. (8)^9,17,18^. The interactions were taken to decay as $\frac{1}{{r}^{\alpha }}$, with the system defined on a lattice in *d*-dimensional space. To give a solvable model, the coefficients ${({B}_{\mu \nu })}_{ij}^{ab}$ were taken to be independent white-noise-correlated random variables. This enabled us to map the operator spreading problem to a stochastic process that incorporates the spatial structure of the interaction, which gives the exact results in Table 1. In ref. ^17^, we argued that general quantum chaotic models with power-law interactions at high energy density would reside in the same universality class as the stochastic model thanks to an effective dephasing of the quantum dynamics.

**Table 1:** The scalings of the local OTOC predicted by the long range Brownian circuit model, see ref. ^17,48,49^

*α*	Light cone	Scaling function	Tail
$\left[\frac{d}{2},d\right)$	$\exp (B{t}^{\eta })$	$C\left(\frac{r}{\exp (B{t}^{\eta })}\right)$	$\frac{1}{{r}^{2\alpha }}$*
*d*	$\exp \left(\frac{{\left(\ln t\right)}^{2}}{4d\ln 2}\right)$	$C\left(\frac{r}{{t}^{\frac{1}{4d}{\log }_{2}t}}\right)$	
$(d,d+\frac{1}{2})$	${t}^{\frac{1}{2\alpha -2d}}$	$C\left(\frac{r}{{t}^{\frac{1}{2\alpha -2d}}}\right)$	
$d+\frac{1}{2}$	$t\ln t$	$C\left(\frac{r}{t\ln t}\right)$	
$(d+\frac{1}{2},d+1)$	*v*_*B*_*t*	$C\left(\frac{r-{v}_{B}t}{{t}^{\frac{1}{2\alpha -2d}}}\right)$	$\frac{1}{{r}^{2\alpha -2d}}*$
*d* + 1	*v*_*B*_*t*	$C\left(\frac{r-{v}_{B}t}{{(t\ln t)}^{\frac{1}{2}}}\right)$	${{{{{{{\rm{erf}}}}}}}}$
$\left[d+1,\infty \right)$	*v*_*B*_*t*	$C\left(\frac{r-{v}_{B}t}{{t}^{\frac{1}{2}}}\right)$	${{{{{{{\rm{erf}}}}}}}}$

Parameters: $B=\frac{d\ln 2}{2{\left(\alpha -d\right)}^{2}}$, $\eta={\log }_{2}\frac{d}{\alpha }$. The tail scalings with * only has numerical support for *d* = 1 along with a few general scaling conjectures.

Returning to the experimental situation with adamantane, we start by normalizing the basic timescales. The nearest neighbor distance between adamantane molecules is 0.67 nm, which yields a value of *J* ~ 2*π* × 410 Hz ~ 2500 Hz frequency for the nearest neighbor dipole coupling (also see ref. ^37^). This translates to a timescale of 0.4 ms. The coherence time during which the data is taken in the experiment is of order 1 ms. We can therefore set *J* ~ 1 and consider about one unit of time. The experimental timescales are currently too short to validate or refute the asymptotic scalings predicted by our theory for *d* = 3 and *α* = 3. Hence, we analyze the short time behavior of the stochastic model.

To do so, we must calibrate the interaction strength in our stochastic model. The dipolar interaction in Eq. (5) has strength *J* when the distance is nearest neighbor distance in adamantane. In the time evolution, this interaction term is non-negligible when *Jt* ~ 1. We take *K*_*ij*_ in Eq. (8) to be $\frac{\sqrt{2K}}{16}\frac{1}{{r}_{ij}^{3}}$. With this choice, the corresponding transition rate generated by a dipolar interaction is $\frac{3}{8}K$. The time scale is thus comparable to the quantum model if we take *K* ~ 1.

We then carry out a Monte Carlo simulation of the stochastic process on the fcc lattice. The results are shown in Fig. 2, where we normalize the time to have a unit of 0.4 ms in our estimation. The fit has two parameters: one is *K*, the other is a time shift. The best fit of the stochastic model to *H*_*DQ*_ experimental data^22^ gives *K* ~ 1.77 and a shift of −0.62 units. The fit is quite close to the experimental data points. One possible interpretation is that we recalibrate the time after local thermalization (the time shift, about 0.87 units in Fig. 2a) beyond which the stochastic approximation is valid. This assumption is subject to test with future experimental data, especially if one can extend the coherence time longer (about 3 units in Fig. 2a). In Fig. 2b, we fit the stochastic model to *H*_*YY*_ experimental data^23^ giving with *K* ~ 2.27 and a shift of −0.79 units. Interpreting the time shift as local thermalization time, in Fig. 2a it is larger. Thus consistently, the fit is not as good as with the larger cluster sizes in Fig. 2a.

## Discussion

We showed that in a chaotic quantum evolution, OTOCs of the global spins are well-approximated by the sum of OTOCs of local spins. When the interaction is local or power law with $\alpha \ge d+\frac{1}{2}$, the global OTOC asymptotically behaves as size of the (linear) light cone, i.e. ${({v}_{B}t)}^{d}$ in *d* dimension. In contrast, when $\alpha \, < \, d+\frac{1}{2}$, the local OTOC’s asymptotic light cone can be super-linear. Assuming the tail distributions in Table 1, the local OTOC scales as ${\left(\frac{r}{{t}^{\frac{1}{2\alpha -2d}}}\right)}^{-2\alpha }=\frac{{t}^{\frac{\alpha }{\alpha -d}}}{{r}^{2\alpha }}$ when $\alpha \in (d,\, d+\frac{1}{2})$. Integration over *r* in *d* dimensions gives a constant factor; the time dependence is ${t}^{\frac{\alpha }{\alpha -d}}$.

When applied to solid adamantane, which corresponds to *α* = *d* = 3, the scaling function is ${\left(\frac{r}{{t}^{\frac{1}{4d}{\log }_{2}t}}\right)}^{-2d}$. This gives an asymptotic time dependence of the global OTOC of ${t}^{\frac{1}{2}{\log }_{2}t}$, which is faster than any power of time. However, on currently accessible timescales, the growth is slower. Up to a cluster size of 10^4^, our numerical simulations of the stochastic model and a two-parameter fit of the resulting data yielded remarkable agreement with the experimental global OTOC curve and give an interpretation of the mysterious *t*^4.36^ power-law fit.

There are several important complications in the comparison with experiments. First, we ignored any effects of dissipation, coupling to the lattice, and so on, which are present. Second, our theory assumes the interaction to be completely random, ignoring approximate or exact global conserved quantities in the dynamics. We expect the the leading operator growth dynamics will not be strongly affected by such slow modes, especially in the orthogonal space of the conserved charges. However, at short times, conserved quantities or closeness to an integrable point could matter. These questions require further research.

The ability to engineer a many-body dipole Hamiltonian and its forward/backward evolution is also accessible in platforms such as dipolar molecules. Existing dipolar molecule experiments have a longer (dimensionless) coherence time but also exhibit a relatively low occupancy of the lattice which hinders operator spreading. We numerically estimate that if the occupancy of each site is modestly increased, say to about 30% or more, then extrapolations of existing experimental configurations should be able to probe the global OTOC dynamics predicted by our theory.

There are a number of other directions for both theoretical and experimental investigations. At lower temperature, the adamantane tumbling is no longer much faster than the dipole coupling time. The random tumbling might then enhance chaos, improving the predictive power of our random interaction model. It would also be interesting to explore the role of dimensionality, e.g., in quasi-one dimensional materials, the role of large values of *M* and conserved quantities by using different compounds.

## Methods

### The diagonal approximation of the global OTOC: random operator estimation

In the expansion of the global OTOC, there are terms that are not diagonal, such as ${{{{{{{\rm{tr}}}}}}}}([{Z}_{a}(t),\, {Z}_{b}][{Z}_{c}(t),\, {Z}_{d}])/{2}^{N}$ with *a* = *c* and *d* ≠ *d*. In this appendix, we give an estimation based on random operators to show that those off-diagonal terms are negligible compared to the sum of the local OTOCs.

Let us consider the case when *a* = *c*, *b* ≠ *d*. The OTOC can be rewritten as

9 $${{{{{{{\rm{tr}}}}}}}}([[{Z}_{a}(t)/{2}^{N/2},\, {Z}_{b}],\, {Z}_{d}] \, {Z}_{a}(t)/{2}^{N/2}).$$

The operator *Z*_*a*_(*t*)/2^*N*/2^ is normalized according to the operator inner product $(A,\, B)={{{{{{{\rm{tr}}}}}}}}({A}^{{{{\dagger}}} }B)$. When expanding it in terms of the Pauli string basis *B*_*μ*_,

10 $${Z}_{a}(t)/{2}^{N/2}=\mathop{\sum}\limits_{\mu }{a}_{\mu }{B}_{\mu },$$

the amplitude squared ∣*a*_*μ*_∣^2^ can be regarded as the probability. At sufficiently long times, the operator *Z*_*a*_(*t*) is scrambled and we assume that it is a random operator supported on *N*_*op*_(*t*) spins, with ${N}_{op}(t) \sim {({v}_{B}t)}^{d}$ in a system with local interactions. We model the effective randomness by treating the *α*_*μ*_ as real random numbers (real because *Z*_*a*_(*t*) is Hermitian). There are ${4}^{{N}_{op}}$ of them, and ∑_*μ*_∣*a*_*μ*_∣^2^ = 1. So the typical size of *a*_*μ*_ is $\sqrt{\frac{1}{{4}^{{N}_{op}}}}={2}^{-{N}_{op}}$.

The double commutator interchanges Pauli strings depending on the operators in the string located at sites *b* and *d*. Strings with *XX* are exchanged with strings with *YY*, and similarly for *XY* and *YX*. Other strings commute with at least one of *Z*_*b*_ and *Z*_*d*_. Writing the corresponding amplitudes as *a*_*μ*_∣_*XX*_, *a*_*μ*_∣_*YY*_, *a*_*μ*_∣_*XY*_, *a*_*μ*_∣_*YX*_, the off-diagonal OTOC is

11 $$8\mathop{\sum}\limits_{\mu }[{{{{{{{\rm{Re}}}}}}}}({a}_{\mu }{|}_{XX}{a}_{\mu }^{*}{|}_{YY})+{{{{{{{\rm{Re}}}}}}}}({a}_{\mu }{|}_{XY}{a}_{\mu }^{*}{|}_{YX})].$$

There are at most ${2}^{{N}_{op}}$ terms in the summation. Assuming they are uncorrelated, the amplitude of the sum is estimated from a random walk to be $\sqrt{{2}^{{N}_{op}}}\times {2}^{-{N}_{op}} \sim {2}^{-{N}_{op}/2}$. Fixing *a* and *b*, there are at most *N*_*op*_ choices of *d*. So the sum of those off-diagonal terms is ${N}_{op}{2}^{-{N}_{op}/2}$, which is negligible for a local OTOC when the two operators are within the butterfly light cone (order 1).

In Supplementary Section III, we refine this argument and do the computation for evolution with a circuit of local gates. The sum of the off-diagonal terms is indeed negligible. Then we argue that the same should hold for long-range interactions. Numerical computations in small systems confirm this observation.

### Diagonal approximation in the presence of conserved quantities

There are two types of diagonal approximations in our effective model: (1) the approximation that only takes the diagonal terms in the expansion of the global OTOC and (2) the approximation that the dynamics of the diagonal terms can be effectively described by a classical stochastic process. Type (2) is also “diagonal" in the sense that we neglect the phases of quantum mechanics thanks to the dephasing from the chaos.

In constrast, the conserved quantities undergoes coherent evolution even in chaotic quantum many-body systems. The Hamiltonians engineered in the NMR experiments in this work have conserved quantities. Both *H*_DQ_ in Eq. (4) and *H*_Y Y_ in Eq. (6) conserve the total energy, and the later also conserves the total *y* spin ∑_*a*_*Y*_*a*_. In the following, we argue that the coherent part of the evolution can not affect the scalings of the global OTOC and both types of diagonal approximations are valid in this sense.

We focus on type (2) first, i.e., the effects of conserved charges on local OTOCs. The operator that spreads in the OTOC is orthogonal to the charge and energy density operators. We evolve the operator ∑_*a*_*Z*_*a*_ while the evolution is given by either *H*_DQ_ in Eq. (4) or *H*_Y Y_ in Eq. (6). An inspection shows that the powers of the Hamiltonian or total *y* spin have zero overlap with the ∑_*a*_*Z*_*a*_ operator. Hence the operator we considered is in an orthogonal space of the conserved charge. The coherent part of the evolution can not affect spreading of ∑_*a*_*Z*_*a*_. In fact according to ref. ^45^, if the evolving operator has non-zero overlap with the charge, then there is a lump in the center that spreads sublinearly (according to the corresponding hydrodynamics of the conserved charge, say diffusion in locally interacting systems). Such lump does not exist if the overlap with the charge is zero. Therefore the conserved charges do not affect our analysis of the local OTOCs.

Moreover, even if there is a non-zero overlap with a (hidden) conserved charge, the charge will undergo a Levy flight with distribution $\frac{1}{{r}^{6}}$ in three-dimension, which is a random walk. Following the analysis of ref. ^45^, the amplitude of the conserved operator in the Pauli basis is conserved, but the probability of those operators decreases as $\frac{1}{{\sqrt{t}}^{d}}$ (for diffusion). Thus the conserved part of the operator carries less and less weight as the evolution proceeds. In the global OTOC, their weight is subleading: The sum of the diagonal terms are (area of local OTOC curve) $\times (1-{{{{{{{\mathcal{O}}}}}}}}({t}^{-d/2}))$. The correction to the diagonal term of the global OTOC is parametrically smaller and can be neglected in large *t*.

Finally, the off-diagonal terms of type (1) are also much smaller than the diagonal terms in an infinite temperature ensemble. For instance, ${{{{{{{\rm{tr}}}}}}}}({e}^{-\beta H}[{Z}_{a}(t),\, {X}_{b}][{Z}_{c}(t),\, {X}_{d}])$ is the thermal average of correlations of the commutators. In a high temperature ensemble, we expect the correlator to factorize:

12 $$\begin{array}{r}{{{{{{{\rm{tr}}}}}}}}({e}^{-\beta H}[{Z}_{a}(t),\, {X}_{b}][{Z}_{c}(t),\, {X}_{d}])\hfill\\ \sim {{{{{{{\rm{tr}}}}}}}}({e}^{-\beta H}[{Z}_{a}(t),\, {X}_{b}]){{{{{{{\rm{tr}}}}}}}}({e}^{-\beta H}[{Z}_{c}(t),\, {X}_{d}]).\end{array}$$

This is justified because in a high temperate ensemble *e*^−*βH*^ has small correlations when the location *a*, *b* are far away from *c*, *d*. Microscopically, one can decompose the time evolved operators *Z*_*a*_(*t*) and *Z*_*c*_(*t*) as a coherent part that propagates diffusively and a ballistic part as in the non-conserving case. The diffusive part is concentrated on local operators with diffusive weight. To generate correlations, both *Z*_*a*_ and *Z*_*c*_ have to diffuse to points *b* and *d*, which lays in the tail of a Gaussian if these locations are separated long apart. Besides, the incoherent part follows the our analysis of the non-conservation case, in which their contribution can be neglected. Therefore the diagonal approximations of type (1) also holds.

**Fig. 3:**
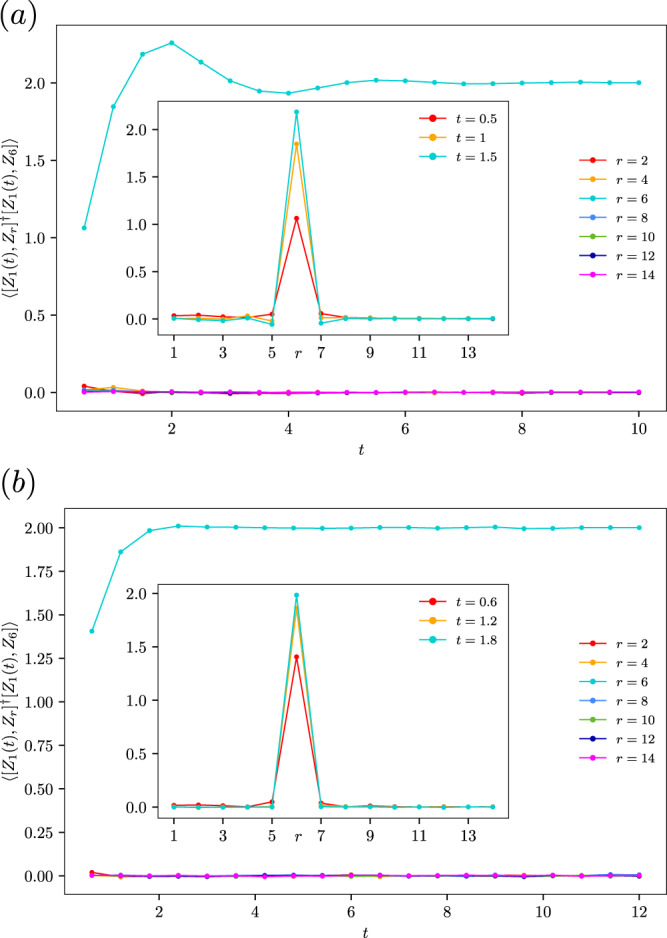
Numerical tests of the diagonal approximation. The local out-of-time ordered commutator (OTOC) ${{{{{{{\rm{tr}}}}}}}}({[{Z}_{1}(t),\, {Z}_{r}]}^{{{{\dagger}}} }[{Z}_{1}(t),\, {Z}_{6}])/{2}^{N}$ for (**a**) evolution by the dipolar Hamiltonian *H*_Y Y_ and (**b**) the 8 $\frac{\pi }{2}$-pulse sequences in the experiments of ref. ^23^ ranging from *r* = 2 (red) to *r* = 14 (magenta). The diagonal OTOC (*r* = 6) is dominant and the growth pattern is similar to the stochastic model prediction. Insets show the spatial profile of the OTOC as a function of *r* from *t* = 1 (red) to *t* = 3 (cyan). The OTOC decays quickly when the index *r* is away from the the diagonal *r* = 6. Source data are provided as a Source Data file.

To provide further evidence, we perform numerical simulations of the OTOCs with the dipolar Hamiltonian *H*_Y Y_. In Fig. 3a, we plot ${{{{{{{\rm{tr}}}}}}}}({[{Z}_{1}(t),\, {Z}_{r}]}^{{{{\dagger}}} }[{Z}_{1}(t),\, {Z}_{6}])/{2}^{N}$ in a one-dimensional open chain of 14 sites. To simulate the effect of *α* = 3 in a three-dimensional chain, we take *α* = 1, which lies at the same critical line of *α* = *d* in our stochastic model. We can see that for different choice of *r*, the diagonal term (*r* = 6) is much larger than the other terms. In the inset, we show the spatial profiles of OTOCs for the first three time steps. The OTOC decays quickly when the index *r* is taken away from the diagonal choice *r* = 6.

We repeat the computation in a more realistic 8 $\frac{\pi }{2}$-pulse setup in ref. ^23^. Ignoring the Zeeman terms, the 8 pulses generate 9 Hamiltonians, which are listed below together with their respective duration

13 $$\begin{array}{l} H_{{{{{{{\rm{ZZ}}}}}}}}\,\, D_1,\, \quad H_{{{{{{{\rm{YY}}}}}}}}\,\, D_2 \, \quad H_{{{{{{{\rm{XX}}}}}}}}\,\, 2D_1 \\ H_{{{{{{{\rm{YY}}}}}}}}\,\, D_2,\, \quad H_{{{{{{{\rm{ZZ}}}}}}}}\,\, 2D_1 \, \quad H_{{{{{{{\rm{YY}}}}}}}}\,\, D_2 \\ H_{{{{{{{\rm{XX}}}}}}}}\,\, 2D_1,\, \quad H_{{{{{{{\rm{YY}}}}}}}}\,\, D_2 \, \quad H_{{{{{{{\rm{ZZ}}}}}}}}\,\, D_1 \end{array}$$

where *D*_1_ and *D*_2_ are

14 $${D}_{1}=\tau (1-\delta )\,\,{D}_{2}=\tau (1+2\delta ).$$

To the first order approximation, the effective Hamiltonian is −12*δτH*_Y Y_ (see Supplemental Material Section I(A) of ref. ^23^). In the experiment, $\delta=[-\frac{1}{2},\, 1]$, *τ* is optimized between 6*μ*s to 16*μ*s. We take $\delta=\frac{1}{2}$. From *J* ~ 2500 Hz, we set *τ* = 0.1. This gives the results in Fig. 3b. The growth of the diagonal OTOC follows the general pattern of the stochastic model. From the inset, the off-diagonal terms are negligible compared to the diagonal terms.

The numerics show that the local OTOCs that have visible magnitudes other than the diagonal terms are those “close” to diagonal terms, for instance when *c* is close to *b* in $-{{{{{{{\rm{tr}}}}}}}}({[{Z}_{a},\, {Z}_{b}]}^{{{{\dagger}}} }[{Z}_{a},\, {Z}_{c}])$. These terms only bring in an overall ${{{{{{{\mathcal{O}}}}}}}}(1)$ correction factors to the global OTOC due to the fast decay when *c* goes away from *b*. In the numerics above, they are at most 5% compared to the diagonal OTOC at *t* = 1, and becomes much less when *t* > 1. Hence our diagonal approximation works well even in Floquet engineered time evolutions in the NMR experiments.

### The Monte Carlo simulation

In this section, we provide details about the Monte Carlo simulations of our stochastic model. We first simulate the values of local OTOCs, which is represented by a discrete height valuable *h* taking integer values among [0, *N*]. For the adamantane molecule, *N* = 16. The height values are defined on each site on the fcc lattice, and *f*(**h**, *t*) is the probability distribution that the height take values as components of **h**. After taking the random averaging, the distribution generated by the Hamiltonian in Eq. (8) satisfies the master equation:

15 $$\frac{\partial f({{{{{{{\bf{h}}}}}}}},\, t)}{\partial t}=	\mathop{\sum}\limits_{j\ne i}\frac{3{D}_{ij}(N-{h}_{i}+1)}{N}{h}_{j}f({{{{{{{\bf{h}}}}}}}}-{{{{{{{{\bf{e}}}}}}}}}_{i},\, t)\\ 	+\mathop{\sum}\limits_{j\ne i}\frac{{D}_{ij}({h}_{i}+1){h}_{j}}{N}f({{{{{{{\bf{h}}}}}}}}+{{{{{{{{\bf{e}}}}}}}}}_{i},\, t)\\ 	-\left\{\mathop{\sum}\limits_{j\ne i}\frac{3{D}_{ij}{h}_{j}(N-{h}_{i})+{D}_{ij}{h}_{i}{h}_{j}}{N}\right\}f({{{{{{{\bf{h}}}}}}}},\, t)$$

where the long range kernel *D*_*ij*_ is $\frac{K}{|i-j{|}^{3}}$.

In the numerics, we set up the fcc lattice embeded in a cubit lattice with *L* ~ 36 in three dimensions. Initially, we set *h* = 1 at the center site and zero elsewhere. We approximate the continuous stochastic process by evolving a small discrete time step Δ*t* ~ 10^−5^ with the rate given by Eq. (15). The rate is $\frac{3}{8}K$ for a site of *h* = 1 site to increase the height of a neighboring site from zero to one. We set *K* = 1. The global OTOC for each instance is the sum of entries of **h** at different sites. We repeat the process several times to obtain an average. In practice, self-averaging effect is quite strong since the system has relatively large *N*.

After obtaining the data, we adjust the values of *K* and the origin of time zero (a linear transformation of the time axis) to fit the experimental curve.

### Polar molecules

We now turn to another physical realization of dipolar interactions via polar molecules and consider the possibility of experiments similar to those in adamantane and other NMR systems. Polar molecules are synthesized by neutral atoms cooled to a few hundred nK. Its rotational groundstate and excited states are taken to be pseudo-spin $\left|\,\,\downarrow \right\rangle$ and $\left|\,\,\uparrow \right\rangle$^13,33,34,46^. For ^40^K^87^Rb molecule, an effective Hamiltonian of the pseudo-spin includes an electric dipolar interaction

16 $$H=\mathop{\sum}\limits_{ij}{D}_{ij}({J}_{\perp }({X}_{i}{X}_{j}+{Y}_{i}{Y}_{j})+{J}_{z}{Z}_{i}{Z}_{j}),$$

where *D*_*ij*_ has been defined in Eq. (5), *J*_*z*_ can be tuned by an applied electric field.

The experimental controls available in the polar molecule case are similar to the NMR setting. The $\left|\,\,\downarrow \right\rangle$ population, or in other words ${{{{{{{\rm{tr}}}}}}}}(\rho Z)$ can be directly measured. Global pseudo-spin rotations can be performed by microwave pulses. These similarities prompt us to propose that global OTOCs can also be probed via polar molecules using a very similar set of pulses as in the nuclear spin experiments. In particular, given an initial state *ρ*, the procedure is to measure the phase rotated quantity ${{{{{{{\rm{tr}}}}}}}}({e}^{i\phi X}{e}^{-iHt}\rho {e}^{iHt}{e}^{-i\phi X}{e}^{-iHt}Z{e}^{iHt})$, and then compute via post-processing its second order derivative with respect to *ϕ*. The result will be proportional to $-{{{{{{{\rm{tr}}}}}}}}([X,\, \rho (t)][X,\, Z(-t)])$, with *ρ*(*t*) = *e*^−*iHt*^*ρe*^*iHt*^ and *Z*( − *t*) = *e*^−*iHt*^*Ze*^*iHt*^, see Supplementary Section I.

Instead of a high temperature ensemble, for polar molecule it is experimentally easiest to begin with a pure state, such as

17 $$\rho (0)=\frac{1}{{2}^{N}}\mathop{\prod}\limits_{i}(1+{Z}_{i}).$$

In the expansion as sum of homogeneous polynomials of *Z*_*i*_, the first term is proportional to ${\mathbb{I}}$, the second term is proportional to *Z* = ∑_*i*_*Z*_*i*_, *etc*.. Truncating to the second term gives us the global OTOC as in the case of nuclear spins. Higher order polynomials of *Z*_*i*_ create extra off-diagonal terms such as

18 $$-{{{{{{{\rm{tr}}}}}}}}([{Z}_{a}(t),\, {Z}_{b}][{Z}_{a}(t),\, {Z}_{c}{Z}_{d}])/{2}^{N}.$$

However, similar to our arguments for the off-diagonal terms, these extra off-diagonal terms to be negligible compared to *C*_*ab*_ in long time, see Methods. So we expect that even the pure state will give the global OTOC, up to an overall constant, at long times.

We now estimate the requirements needed to probe the long-time regime in experiments with KRb molecules. In this case, *J*_⊥_ is about 2*π* × 104 ~ 650 Hz. The coherence time shown in the Ramsey spectroscopy experiment is of order 10 ms. Hence the coherence time is about 10 units of time, an order of magnitude larger than the nuclear spin experiment. However, unlike in the nuclear spin case, experimental realizations to date involve a dilute lattice of spins, with many lattice sites empty.

Previous experiments achieved a filling factor of less than 10%, which is in sharp contrast to the 16 spins on each site in the nuclear spin experiment. As expected, the low occupancy significantly hinders the spreading, although the long-range nature of the dipolar interaction moderates this slowdown to some extent. To give a crude estimate, imagine a sphere surrounding one molecule. The volume of the sphere is $\frac{4\pi }{3}{r}_{0}^{3} \, \approx \, 4{r}_{0}^{3}$. Taking the occupancy to be 5%, a volume of $4{r}_{0}^{3}=20$ has only one site occupied by a molecule. On average, the nearest neighbor interaction is reduced by a factor of $\frac{1}{{r}_{0}^{6}}=\frac{1}{25}$ (in the classical stochastic model, the rate is $\frac{1}{{r}^{6}}$ rather than $\frac{1}{{r}^{3}}$ due to dephasing). Hence 10 units of time can only populate a cluster of size $10/25\times \frac{3}{4}=0.3$, which is barely one spin. Keeping with this estimate, the linear size of the cluster is $10\times {(4p)}^{2}\times \frac{3}{4}=120{p}^{2}$. The volume is 120^3^*p*^6^. Thus the thresholds of occupancy to reach cluster sizes of 10, 10^2^, 10^3^ are 13.4%, 19.7% and 28.9% respectively.

**Fig. 4:**
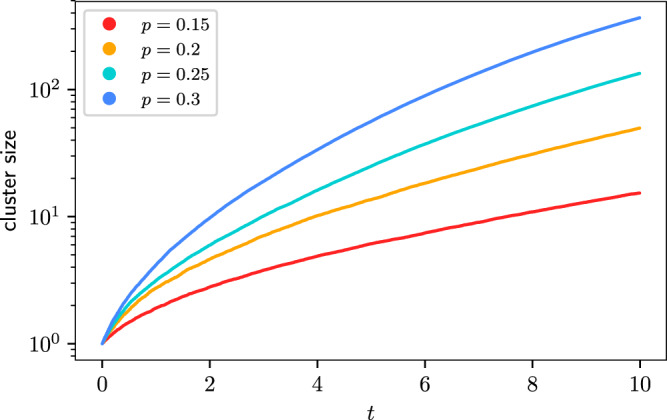
Stochastic model predictions of global OTOC for KRb polar molecule. Parameter *p* is the occupation fraction from 0.15 (red) to 0.3 (blue). Source data are provided as a Source Data file.

We numerically simulate the stochastic process on a simple cubic lattice for *p* ∈ [15%, 30%]. The global OTOC does match the order of magnitude of our estimation, see Fig. 4

In summary, the coherence time (in units where *ℏ* = 1) of the dipolar molecule systems is roughly one order of magnitude larger than in the NMR system, but the relatively low density of occupation hinders rapid operator growth. However, the cluster size has a 6th power dependence with respect to the occupancy *p* and 3rd power dependence with respect to the coherence time. Hence, reaching a cluster of size 10^3^ requires only a moderate increase in occupancy or coherence time. Assuming the presently available factor of 10 enhancement in the coherence time, we estimate the threshold to see significant operator growth to be *p* ≈ 30%. Beyond this density, one should be able to observe some of the growth patterns of the global OTOC in the polar molecule system.

### Computations of off-diagonal OTOCs

We provide further evidence that off-diagonal OTOCs are negligible in a variety of models. For example, for holographic CFTs one can extend the results of ref. ^2^ by mapping off-diagonal OTOCs at non-zero energy density to certain two-sided correlations in a black hole spacetime where the operators are inserted at different spatial locations. Using, for example, a geodesic approximation to the correlator, one can then verify that off-diagonal OTOCs decay exponentially with the separation between operators. We can also study this question in a variety of lattice models using exact diagonalization and Krylov techniques.

To illustrate the basic physics, we consider a spin model, studied at finite size using exact evolution of the many-body quantum state. The model is a long-range version of the well studied kicked Ising model. It is a Floquet model with a single period of time evolution generated by *U* = *U*_*I*_*U*_*K*_ with

19 $${U}_{K}=\exp \left(ib\mathop{\sum}\limits_{r}{\sigma }_{r}^{x}\right)$$

and

20 $${U}_{I}=\exp \left(iJ\mathop{\sum}\limits_{r,d}\frac{1}{{d}^{\alpha }}{\sigma }_{r}^{z}{\sigma }_{r+d}^{z}+i\mathop{\sum}\limits_{r}{h}_{r}{\sigma }_{r}^{z}\right).$$

The couplings *h*_*r*_ are random and drawn from a Gaussian distribution with mean zero and standard deviation *h*.

We choose this model because in the local case it is a model of strong quantum chaos^47^. In particular, when *α* = *∞* (local interactions) and *J* = *b* = *π*/4, the model is at the dual unitary point and exhibits a number of exact features characteristic of quantum chaos.

**Fig. 5:**
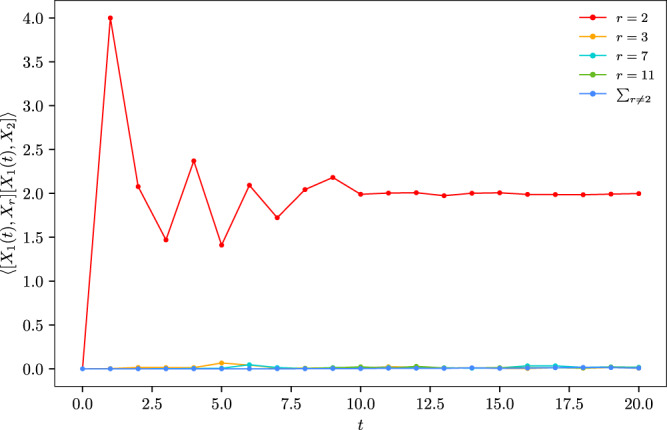
Numerical comparisons for the diagonal and off-diagonal terms. Exact computation of $|\left\langle [{X}_{1}(t),\, {X}_{r}(t)][{X}_{1}(t),\, {X}_{2}(t)]\right\rangle|$ in the long range kicked Ising model (Eq. (20)) of linear size *L* = 14. *X*_*i*_(*t*) is the local Pauli *X* operator at site *i* and time *t*. *r* ranges from 2 (red) to 11 (green). Blue curve represents the sum of all off-diagonal terms. Source data are provided as a Source Data file.

Here we consider a long-range version of the model, still with *J* = *b* = *π*/4 and now with *α* < *∞*. As a simple diagnostic, we compute

21 $$ | \langle [{X}_{1}(t),\, {X}_{r}(t)][{X}_{1}(t),\, {X}_{2}(t)]\rangle |,$$

where the quantum average is taken over a random state in Hilbert space. This would reduce to a trace in the maximally mixed state if we also averaged over the choice of random state, but these data are for a single realization of the random state. The diagonal term corresponds to *r* = 2, which gives order 1 value; the off-diagonal terms and their sum is two orders of magnitude smaller, see Fig. 5. This indicates the OTOCs of global operators can be approximated by diagonal OTOCs of local operators, which is interpreted as the area under the local OTOC curve.

## Supplementary information

Supplementary Information

## Data Availability

Source data are provided with this paper.

## Source data

Source Data

## Code availability

The computer code to simulate the stochastic process is available upon reasonable request.
