# Supplementary material for: Operator growth from global out-of-time-order correlators
Source: Nat Commun. 2023 Jun 9;14:3411. doi: 10.1038/s41467-023-39065-5 (PMC10256796; doi:10.1038/s41467-023-39065-5)
Supplement: Supplementary file 1 — Supplementary Information [file 41467_2023_39065_MOESM1_ESM.pdf]

Supplementary Information

Tianci Zhou[1,2,*] and Brian Swingle[3]
[1]Kavli Institute for Theoretical Physics, University of California, Santa Barbara, CA 93106, USA
[2]Center for Theoretical Physics, Massachusetts Institute of Technology, Cambridge, Massachusetts 02139, USA
[3]Brandeis University, Waltham, MA 02453, USA
(Dated: May 15, 2023)

I. NMR EXPERIMENTS REVIEW

Nuclear magnetic resonance (NMR) is a standard technology that uses nuclear spin as the degree of freedom to study interacting quantum magnetism in and out of equilibrium. In this section, we review at a high level the experimental procedures to measure the global OTOC in materials like adamantane and some standard theoretical interpretations of the data. Throughout the section, we use $I_{iz} = \frac{1}{2}Z_i$ to represent the nuclear spin-$\frac{1}{2}$ operator at site i, and $I_z = \frac{1}{2}\sum_i Z_i$ for the total spin operator.

A. The single quantum coherence

In a typical solid state NMR experiment, a material is exposed to a strong uniform magnetic field. The energy scale of this Zeeman interaction is much larger than any other scale in the problem except the temperature, hence in equilibrium the nuclear spins are polarized in the z direction—the direction of the magnetic field. For proton nuclear spin

$$H_{\text{Zeeman}} = -\gamma I_z B_0 = -\gamma \frac{1}{2} Z B_0 \tag{1}$$

where γ is the gyromagnetic ratio. If B_0 is $1T$, γB corresponds to $2\pi \times 42.6$ M Hz or $2mK$ in energy. At room temperature, the initial density matrix at thermal equilibrium can be expanded in the high temperature limit

$$\rho = \frac{e^{+\frac{\gamma B_0}{2k_B T} Z}}{\text{tr}(e^{+\frac{\gamma B_0}{2k_B T} Z})} \propto \mathbb{I} + \frac{\gamma B_0}{2k_B T} Z \tag{2}$$

Since in the correlators below, the $\mathbb{I}$ part of the density matrix gives zero contribution, oftentimes the density matrix is written as $\rho = Z$.

Modulated radio-frequency waves exert a magnetic field in the x direction on top of the Larmor precession. When the radio-frequency wave is removed, the magnetization will decay to its equilibrium value through spin-spin or spin-lattice relaxation processes. The x, y magnetization can generate induction in the coil, and reading out the free induction signal can tell us $\text{tr}(\rho X)$ and $\text{tr}(\rho Y)$. The measurement of $\text{tr}(\rho Z)$ can be converted to the X, Y magnetization by first imposing a spin rotation pulse—the $\frac{\pi}{2}$ pulse—and measuring the free induction signal afterward.

There are internal interactions, on the scale of kHz. The most prominent one for protons is the dipole interaction. But since the Zeeman field corresponds to an energy scale of 10^3 kHz, the dipole interaction is well-approximated by the secular form,

$$H_{\text{int}} = \sum_{i \neq j} D_{ij}(3I_{zi}I_{zj} - \mathbf{I}_i \cdot \mathbf{I}_j), \tag{3}$$

where

$$D_{ij} = \frac{\gamma^2 \hbar}{r_{ij}^3} \frac{3\cos^2\theta_{ij} - 1}{2}. \tag{4}$$

Other interactions, such as the chemical shift, scalar coupling and quadrupole coupling either vanish for protons or are much smaller.

With this setup, one can measure the magnetization of the time evolved state, for example

$$\text{tr}(e^{iHt}\rho e^{-iHt} X) \tag{5}$$

Since the X operator changes the total Z eigenvalue by ± 1, the measurement only probes the matrix elements of $\rho(t)$ slightly away from the diagonal. Hence, it is called the single quantum coherence.

B. Multiple quantum coherence

The multiple quantum coherence corresponds to the expectation values of operators that change the total Z eigenvalue by more than 1. We can systematically decompose the density matrix as

$$\rho = \sum_n \rho_n, \tag{6}$$

where the n-quantum coherence component satisfies

$$e^{i\phi I_z} \rho_n e^{-i\phi I_z} = \rho_n e^{in\phi}. \tag{7}$$

Formally, MQC can be defined as

$$g_n = \frac{1}{\text{tr}(I_z^2)} \text{tr}(\rho_n \rho_{-n}) \tag{8}$$

* tzhou13@mit.edu

Experimentally, one can add a twist after a time evolution to measure the Fourier transform of the multiple quantum coherence.

$$I(\phi,t)=\frac{1}{\mathrm{tr}(I_z^2)}\mathrm{tr}(e^{i\phi I_z}\rho(t)e^{i\phi I_z}e^{iHt}I_z e^{-iHt}) \tag{9}$$

In fact, expanding $\rho(t)$ and using the property in Eq. (7), we have

$$I(\phi,t)=\frac{1}{\mathrm{tr}(I_z^2)}\mathrm{tr}(\sum_n \rho_n e^{in\phi}\sum_m \rho_m)=\sum_n g_n e^{in\phi}. \tag{10}$$

On the other hand

$$\begin{aligned}\sum_n n^2 g_n &= -\partial_\phi^2 I(\phi,t)\Big|_{\phi=0}\\ &=\frac{1}{\mathrm{tr}(I_z^2)}\mathrm{tr}([I_z,[I_z,\rho(t)]]I_z(t)) \quad (11)\\ &=-\frac{1}{\mathrm{tr}(I_z^2)}\mathrm{tr}([I_z,I_z(t)][I_z,I_z(t)]).\end{aligned}$$

Therefore if we sample $I(\phi,t)$ at discrete values of ϕ, we can do an inverse Fourier transform to figure out the multiple quantum coherence g_n, whose second moment is the global OTOC.

C. Engineering of the backward time evolution

With the presence of the external radio-frequency wave, the total Hamiltonian in the rotating frame is

$$H=H_{\mathrm{int}}+H_{\mathrm{rf}}(t) \tag{12}$$

in which the latter can be time dependent.

The analysis is usually carried out in the toggling frame. Define $U_{\mathrm{rf}}(t)=\mathcal{T}e^{-i\int_0^t H_{\mathrm{rf}}(t')dt'}$, the toggling frame Hamiltonian is defined as

$$H_{\mathrm{tf}}(t)=U_{\mathrm{rf}}^\dagger(t)H_{\mathrm{int}}U(t)_{\mathrm{rf}} \tag{13}$$

so that

$$\mathcal{T}e^{-\int_0^t H(t')dt'}=U_{\mathrm{rf}}(t)\mathcal{T}e^{-\int_0^t H_{\mathrm{tf}}(t')dt'} \tag{14}$$

If the pulse is periodic, then $U_{\mathrm{rf}}=1$ at those periods. So if we make measurements at those time points, the evolution is determined by the toggling frame Hamiltonian. The time independent effective Hamiltonian can be worked out by a Magus expansion. At the lowest order, the effective Hamiltonian is the average of the toggling frame Hamiltonian

$$H_{\mathrm{eff}}=\frac{1}{T}\int_0^t H_{\mathrm{tf}}(t')dt' \tag{15}$$

This is the basis to engineer interacting Hamiltonians in the NMR system.

In the 80s, pulse sequences with four $\frac{\pi}{2}$ pulses were used to transform the original dipolar Hamiltonian to the double quantum Hamiltonian

$$H_{\mathrm{DQ}}=\sum_{ij}D_{ij}(X_iX_j-Y_iY_j). \tag{16}$$

Since the double quantum Hamiltonian is an operator of second order quantum coherence, a rotation by π can create a minus sign. Thus an additional π pulse on top of the original pulse sequence can create $-H_{\mathrm{DQ}}$, enabling backward time evolution.

The experiment in the main text that we cite used a different approach. It is an eight pulse sequence with parameter δ in the time interval of each pulse. It can create the dipolar Hamiltonian in Y direction with strength proportional to δ,

$$H_{\mathrm{YY}}=\delta\sum_{ij}D_{ij}(Y_iY_j-Z_iZ_j-X_iX_j) \tag{17}$$

Thus, by changing the sign of δ, which amounts to changing the time interval between the pulses, one can obtain $-H_{\mathrm{YY}}$ and the backward time evolution.

It is therefore technically possible to measure $I(\phi,t)$ in Eq. (9) in an experiment.

D. The Weakly Polarized State Approximation

Before moving on, let us comment on one approximation used in the above analysis. In the NMR setup to measure the OTOC, one of the time evolved operators $Z(t)$ comes from the high temperature expansion of the time evolved density matrix $\rho(t)$. In a Zeeman field in the z direction, the initial density matrix can be expanded as

$$\rho(0)\sim e^{-\frac{\frac{\gamma}{2}BZ}{k_BT}}\sim \mathbb{I}-\frac{\gamma BZ}{2k_BT}, \tag{18}$$

where $\mathbb{I}$ is omitted later in the calculation, resulting in the schematic $\rho(t)\sim Z(-t)$.

Since Z is a many-body operator, this expansion is formally only valid if the system size is sufficiently small. The gyro-magnetic ratio in 1 T magnetic field is about $2.4mK$ for the protons in adamantane. At room temperature, the (dimensionless) coefficient in front of Z is of order 10^{-5}. Hence the expansion is valid when the operator norm of Z is smaller than 10^5. This sets an upper limit on the cluster size (the global OTOC).

When the scale of the global OTOC is beyond 10^5, the weakly polarized state assumption in Eq. (18) fails, and one needs to consider the high temperature expansion for each spin separately,

$$\rho(0)\sim\prod_i\left(\mathbb{I}-\frac{\gamma BZ_i}{2k_BT}\right), \tag{19}$$

which contains higher order monomials of Z_i. This is closer to the situation encountered in the polar molecule

setup in Methods, the initial state is typically a polarized pure state.

II. MULTIPLE COHERENCE MEASUREMENT IN ADAMANTANE: THE KN SPACE APPROACH

In this work, we choose the material adamantane as an example, for both its long history in the NMR community and because recent global OTOC data is available. Adamantane is a solid polycrystal at room temperature. The crystal structure is face-centered cubic (fcc) with one adamantane molecule ($C_{10}H_{16}$) at each lattice site. The Hydrogen protons comprise the active nuclear spins, so there are 16 spin-1/2s per lattice site. Researchers can engineer a double quantum Hamiltonian

$$H_{\mathrm{DQ}} = \sum_{a\neq b} D_{ab}(X_a X_b - Y_a Y_b), \tag{20}$$

or a dipolar Hamiltonian

depending on the pulse sequences. Adamantane also has the peculiar feature that the molecules tumble in place in the lattice at relevant temperatures due to their nearly spherical nature. This enables us to simplify the interaction further in the main text.

The measurement of global OTOCs in adamantane molecules dates back to the 1980s under the name of multiple quantum coherences[1–7], although at that time only a handful of coherent spins were involved [8]. More recently, thanks to improved coherence times[7] and the scaled Hamiltonian technique[9], the number of coherent spins can be as large as 10^4 [10]. Loschmidt echoes, a related class of observables that also probe time reversal effects, have also been studied [11].

Next, we review the popular Kn space approach [8] adopted by the NMR community to understand multiple quantum coherence. This approach does not take into account the spatial structure of the interaction, and not surprisingly, it predicts an exponential growth of the global OTOC in time. As an alternative to this approach, we apply our theory to a simple stochastic model to estimate the global OTOC.

A. The Kn space approach

The experiments of interest do not measure the global OTOC directly, but rather extract it from multiple quantum coherences (MQC) defined as follows [6]. Let ρ be the time evolved operator $\rho = Z(t)$. In NMR, this represents the density matrix neglecting the identity part that does not participate in the dynamics (see Sec. I and the discussion in Sec. I D). The operator Z appears because at high temperature in thermal equilibrium the sample is weakly polarized due to the Zeeman field.

The density operator ρ can be decomposed as

$$\rho = \sum_n \rho_n, \tag{21}$$

where ρ_n increases the total spin z quantum number by n. Operators like X and Y change the total spin z quantum number by ± 1. They are an example of a single quantum coherence. When $|n| > 1$, $\rho_n \neq 0$ are called multiple quantum coherences, as they indicate the structure in density matrix further away from the diagonal. Formally, the operator ρ_n satisfies

$$e^{i\phi Z}\rho_n e^{-i\phi Z} = \rho_n e^{in\phi}, \tag{22}$$

and the "intensity"

$$g_n = \frac{1}{\mathrm{tr}(ZZ)}\mathrm{tr}(\rho_n \rho_{-n}) \tag{23}$$

defines the multiple quantum coherence. The second moment of the MQC is proportional to the global OTOC[12](see Sec. I),

$$\sum_n n^2 g_n = -\frac{1}{\mathrm{tr}(ZZ)}\mathrm{tr}([Z, Z(t)]^2). \tag{24}$$

So far what we have said in this subsection is general. Next, we review some standard intuition for the MQC based on a simple counting argument. A n-quantum coherence maps a state with x up spins to states with $n+x$ up spins. For a system of K spins, there are

$$\sum_{x=0}^{K}\binom{K}{x}\binom{K}{n+x} = \binom{2K}{K+n} \sim 2^{2K}\exp\left(-\frac{n^2}{K}\right) \tag{25}$$

operators that belong to the n-quantum coherence space. If all these operators are equally likely, then g_n will be roughly a Gaussian function, $g_n \sim \exp(-\frac{n^2}{K})$. Therefore, the second moment of g_n, the global OTOC, will scale as K, the number of spins in the system.

In the dynamical setting, this idea is generalized by allowing K to be a function of time. $K(t)$ represents the effective size of the spin cluster supporting the n-quantum coherence operators. In practice, $K(t)$ is obtained by a Gaussian fit from the MQC. Hence, the fluctuations of the MQC as a function n indicate the dynamically growing cluster size and give the growth of the global OTOC. Our result above in essence shows that $K(t) \propto \int d^d r C(r,t)$, where $C(r,t)$ is the corresponding local OTOC.

One approach to model the dynamics of $K(t)$ is to replace the full quantum dynamics with a stochastic process in the Kn plane [8]. The transition rate between two points in the Kn plane is taken to be proportional to the number of interaction terms that cause such a transition. We use the transition rates in Ref. 8 and reproduce the results for the double quantum Hamiltonian in Supplementary Figure 2(a) and Supplementary Figure 2(b) in the section.

However, this particular stochastic approach does not take the spatial dependence of the interactions into account. It implicitly treats all the sites on an equal footing. Hence, we expect this model to show an exponential growth of the OTOC, a phenomenology common to systems with all-to-all interactions. We verify this expectation in Supplementary Figure 1[1]. Note that when this approach was proposed, the experimentally accessible system sizes were relatively small ($N \lesssim 21$) and the model compared favorably with data.

In addition to the stochastic Kn approach, there are also other non-stochastic effective models such as the Levy-Gleason model and recent variants[15, 16]. In most of these models, the locality of the interaction is not incorporated. As experiments push to larger sizes and longer times, the spatial structure of the interactions becomes important.

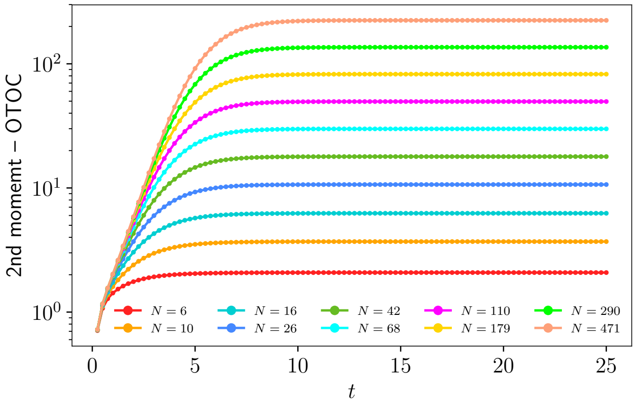

Supplementary Figure 1: The global OTOC computed from the stochastic motion in the Kn space approach for $N \leq 600$. Without the spatial struction of the interaction, the curve takes an exponential growth before saturation.

B. Transition rates and detailed Kn space calculation for dipolar Hamiltonian

On top of the multiple coherence decomposition in Eq. (21), each multiple quantum coherence component of the density matrix is further decomposed as

$$\rho_n = \sum_K \rho_{Kn}. \tag{26}$$

Using the Pauli string basis, the number K here is the number of Pauli operators in the string. In Sec. II A, we introduced this number as the effective size of the spin system. It is generically time dependent. The time evolution will transfer the operator from a smaller K to large K. One can then view this as a stochastic process in the Kn space, where the transition probability is determined by the number of interaction terms connecting the states. The assumption here is that all states with the same K and n are equally likely and the transition can occur when the Hamiltonian allows. A finer multiple quantum coherence, or the probability of staying at state K, n is given by g_{Kn}. Clearly, $\sum_K g_{Kn} = g_n$.

Ref. 8 used the double quantum Hamiltonian as an example. Defining

$$Q_{Kn} = \sum_{c_+=n}^{K} \binom{K}{c_+}\binom{K-c_+}{c_+-n}, \tag{27}$$

the transition probability can be written as

$$\begin{aligned} W_{K+1,n\pm2,Kn} &= \frac{K(N-K)}{N-1}\frac{Q_{K-1,n}+Q_{K-1,n\pm1}}{Q_{Kn}} \\ W_{K-1,n\pm2,Kn} &= \frac{K(K-1)}{N-1}\frac{Q_{K-2,n\pm2}+Q_{K-2,n\pm1}}{Q_{Kn}} \end{aligned} \tag{28}$$

where N is the total number of spins.

We simulate this process and reproduce the multiple quantum coherence for $N = 6$ and 21 sites, see Supplementary Figure 2(a) and Supplementary Figure 2(b).

When we increase the number of sites to a few hundred, we observe that the OTOC grows exponentially in time (Supplementary Figure 1). Hence the Kn space model, which ignores the spatial structure of the interactions, gives exponential growth of the OTOC.

III. THE DIAGONAL APPROXIMATION OF THE GLOBAL OTOC: QUANTUM CIRCUITS ESTIMATION

In the main text, we argued that the global OTOC can be well approximated by the diagonal terms—the local OTOCs:

$$\begin{aligned} -\mathrm{tr}([Z(t), Z]^2) &= -\sum_{abcd} \mathrm{tr}([Z_a(t), Z_b][Z_c(t), Z_d]) \\ &\approx -\sum_{ab} \mathrm{tr}([Z_a(t), Z_b][Z_a(t), Z_b]). \end{aligned} \tag{29}$$

We thus conclude that the global OTOC measures the area under the local OTOC curve.

In this section, we present a more rigorous calculation to show why the off-diagonal terms can be neglected for the sake of estimating the global OTOC scaling.

In the following, we analyze the diagonal and off-diagonal terms in the setup of a quantum circuit with local unitary gates. Typically, random averaging over [17–22] over those unitaries provides way to compute local OTOCs in terms of the free energy of Ising domain

[1] Other variants that take the distribution to be a superposition of Gaussian functions with different cluster sizes also predict exponential growth [13, 14].

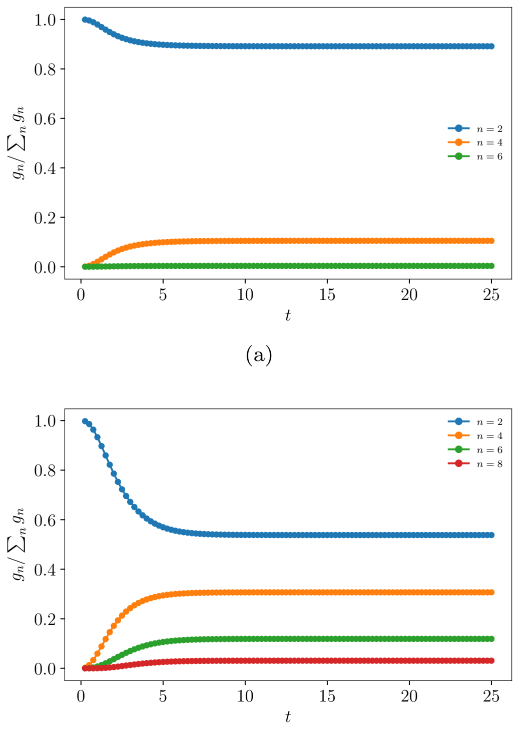

(a)

(b)

Supplementary Figure 2: Numerical results of the normalized multiple quantum coherence $g_n/\sum_n g_n$ for (a) $N=6$ and (b) $N=21$ spins.

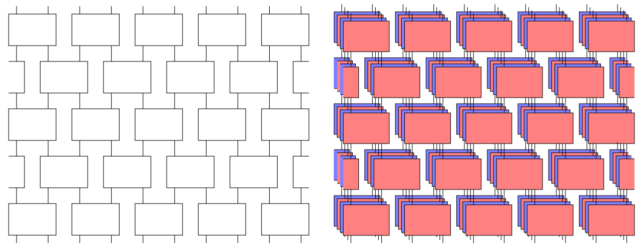

(a) (b)

Supplementary Figure 3: Unitary evolution matrix in the form of a quantum circuit. (a) The structure of the circuit. The four-leg tensor is a unitary matrix (gate) on two sites. The structure models local interactions. (b) The forward and backward evolutions involved in the computation of OTOC. Red/blue gates represent forward/backward unitary evolution.

walls. Here we use the entanglement membrane picture developed in Ref. 17, which enables us to estimate the local OTOCs without random averaging. Thus we expect the estimation to hold for a generic chaotic evolutions with or without disorder.

This calculation justifies to neglect the off-diagonal OTOC terms for locally interacting chaotic systems. We then generalize the estimations to systems with long-range interactions.

A. Systems with local interactions

We model the time evolution for systems with local interactions via a quantum circuit with the architecture shown in Supplementary Figure 3(a). Our system is chaotic, so we take a generic choice of gates such that the whole evolution is not integrable.

There are four terms in the expansion of the global OTOC in Eq. (29)

$$\begin{aligned}&\mathrm{tr}([Z_a(t),Z_b][Z_c(t),Z_d]) =\\ &-\mathrm{tr}(Z_a(t)Z_bZ_c(t)Z_d)+\mathrm{tr}(Z_a(t)Z_c(t)Z_dZ_b)\\ &+\mathrm{tr}(Z_a(t)Z_bZ_dZ_c(t))-\mathrm{tr}(Z_a(t)Z_dZ_c(t)Z_b).\end{aligned} \tag{30}$$

We represent each term in a tensor network diagram as shown in Supplementary Figure 3, where red box represents forward evolution and blue box represents backward evolution. At sites other than a,b,c and d, we put $\cup\ \cup$ at the bottom of the tensor in Supplementary Figure 3 and $\frown$ on the top: those just implements the matrix product between operators and the trace. At a, b, c and d, we insert operators Z_a, Z_b, Z_c and Z_d at the appropriate time slices (bottom to time 0, and top to be time t).

For example, at site a/c, we have

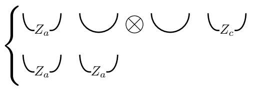

$a \neq c$, $a = c$. (31)

They are connected with the tensor in Supplementary Figure 3 at the bottom.

At b/d, we have

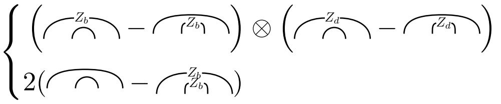

$b \neq d$, $b = d$. (32)

They connect with the tensor in Supplementary Figure 3 on the top.

We then use the idea from Ref. 17 and convert each term into a statistical mechanical problem of interacting spins.

Next we set up the notations and review the mapping to the spin model without random averaging. The goal is to replace the four copies of the gate at the same space time locations of Supplementary Figure 3 (i.e. $u\otimes u^*\otimes u\otimes u^*$) by three choices of spins: $+$, $-$ and $\perp$, and list rules for their interactions. We assume the local Hilbert space dimension to be q (for spin-$\frac{1}{2}$ $q=2$).

There are four copies of the local Hilbert space in the structure $u\otimes u^*\otimes u\otimes u^*$. We define two tensors in among Hilbert spaces as

$$|+\rangle = |\cup\ \cup\rangle \quad |-\rangle = |\smile\!\!\smile\rangle, \tag{33}$$

where the graphical notation means a delta function between the corresponding local Hilbert spaces, in analogous to the boundary conditions we listed above. These states are not orthonormal, thus we have the dual basis

$$
\begin{aligned}
|+^*\rangle &= \frac{1}{q^2-1}(|+\rangle - \frac{1}{q}|-\rangle) \\
|-^*\rangle &= \frac{1}{q^2-1}(|-\rangle - \frac{1}{q}|+\rangle)
\end{aligned} \tag{34}
$$

such that $\langle +^*|+\rangle = \langle -^*|-\rangle = 1$, and $\langle +^*|-\rangle = \langle -^*|+\rangle = 0$. For states defined on two sites, the dual basis are

$$
\begin{aligned}
|(++)^*\rangle &= \frac{1}{q^4-1}(|++\rangle - \frac{1}{q^2}|--\rangle) \\
|(++)^*\rangle &= \frac{1}{q^4-1}(|--\rangle - \frac{1}{q^2}|++\rangle)
\end{aligned} \tag{35}
$$

We can similarly construct the kets $\langle ++|$ and $\langle --|$ from $\langle \frown \frown|$ and $\langle \frown|$, and so are their dual basis.

With these facilities, the random averaging of $u \otimes u^* \otimes u \otimes u^*$ can be written as

$$
\overline{u \otimes u^* \otimes u \otimes u^*} = |(++)^*\rangle\langle ++| + |(--)^*\rangle\langle --| \tag{36}
$$

In Ref. 17, we generalize this expression to systems without randomness, so that there is an additional term in this expression

$$
u \otimes u^* \otimes u \otimes u^* = |(++)^*\rangle\langle ++| + |(--)^*\rangle\langle --| + \text{"} \perp \text{"} \tag{37}
$$

Here the $\perp$ state is the difference of the LHS and the first two terms of the RHS. It is a tensor that depends on the gate u. Each gate in the circuit then has three choices, $+$, $-$ and $\perp$ according to the terms in Eq. (37), see Supplementary Figure 4 (a). The OTOC then becomes a partition function of those spins on each gate, and the boundary conditions are given above (see a change of convention in Eq. (38) and Eq. (39) below).

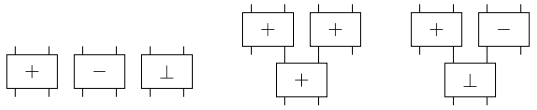

Supplementary Figure 4: (a) Three choices of spins according to the decomposition in Eq. (37). Rules of the spins: (b) The same spins on the top two gates forces the same spin below them (the weight is 1) (c) $\perp$ spin can only occur at the domain wall – below different spins or another $\perp$ spins.

The expression on the LHS of Eq. (36) is time reversal invariant, while the RHS is not (explicitly) due to a choice of the the non-orthogonal states $++$ and $--$. For later convenience, we adopt a slightly different convention and turn the whole tensor upside down, with a and c on the top, b and d at the bottom. To avoid confusion, we rewrite the boundary condition at a/c as

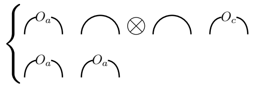

$a \neq c$; $a = c$ (38)

and at b/d as

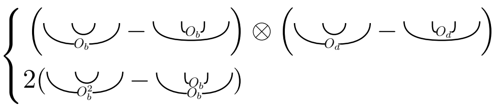

$b \neq d$; $b = d$. (39)

We have write with the more general traceless operators $O_{a,b,c,d}$ here.

The rules for the spin assignment is restrictive due to unitarity and causality. When the spins of neighboring gates are the same, then it forces the gate below to have the same spin. Such a structure has weight 1. When the spins of neighboring gates are different, the spin below can be either $+$, $-$ or $\perp$. The first two choices form a perfect domain wall, which has weight $\frac{q}{q^2+1}$ (for spin-$\frac{1}{2}$, it is $\frac{2}{5}$). The $\perp$ spin can only occur beneath a domain wall or other $\perp$ spins, see examples in Supplementary Figure 4 (b) and (c). Those rule indicate that this is an ordered phase: domain wall at the boundary induces domain walls inside the bulk. The $\perp$ spins can perturbatively dress the domain wall to have order 1 width.

We now discuss the spin configurations and the associated scaling for different choice of a, b, c and d.

Case 1: local OTOC: $a = c$, $b = d$

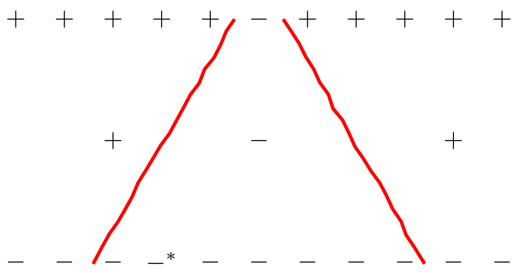

Supplementary Figure 5: Domain wall configuration for $a = c$, $b = d$. The operator insertion point a at the bottom forces a $-$ spin. There are two domain walls emitted from the top.

The scalings of a local OTOC has been analyzed in Ref. 17. We review the calculation and set up a benchmark for the other off-diagonal terms.

Consider a local OTOC with general traceless operator O_a and O_b

$$
-\frac{1}{2}\mathrm{tr}([O_a, O_b]^2) \tag{40}
$$

The boundary conditions are

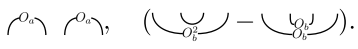

(41)

To simplify the result (and remove the unimportant dependence on the choice of the operators), we can random

average the single site traceless operator $O_a \to V_a O_a V_a^\dagger$. This amounts to contract $|+^*\rangle\langle+| + |-^*\rangle\langle-|$ with the boundary loops at site a and site b. The state at site b becomes

$$q\mathrm{tr}(O_b^2)|-^*\rangle \tag{42}$$

i.e. the spin at b will force a $-$ spin above it. Then the top site a must have be a $-$ spin, otherwise an all $+$ boundary condition can be pushed to the bottom with unitarity property and the whole quantity vanishes. So we have

$$\frac{\mathrm{tr}(O_a^2)}{q^2-1}\langle-| \tag{43}$$

on the top.

In summary, the top boundary has two domain walls emitted at the two sides of a. The bottom boundary condition favors $-$ spins, so the two domain walls tend to expand to a large $-$ domain. However domain wall with larger slope (here defined to be horizontal distance divided by vertical distance, which has the dimension of velocity) cost more energy. The equilibrium is reached when both of the domain walls are stretched as slope v_B (the quantum butterfly velocity), see Supplementary Figure 5. The domain wall fluctuates within a region of size $\sqrt{t}$. When site b is outside the slope v_B of site a, it will then force the domain wall to have slope larger than v_B, resulting in the exponential decay of the OTOC.

Case 2: $a = c$ and $b \neq d$

The analysis for $a \neq c$ and $b = d$ is the identical, because

$$\begin{aligned}&\mathrm{tr}([Z_a(t), Z_b][Z_c(t), Z_d]) \\ =&\mathrm{tr}([Z_a, Z_b(-t)][Z_c, Z_d(-t)]).\end{aligned} \tag{44}$$

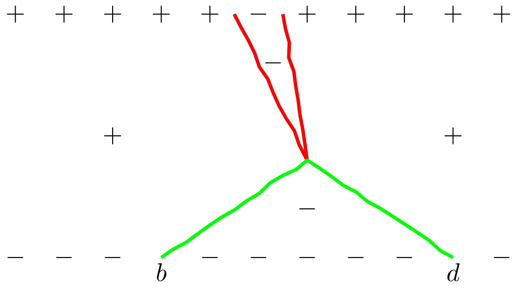

Supplementary Figure 6: Domain wall configuration for $a \neq c$, $b = d$. There are $\perp$ clusters connecting a, b, c, d.

The boundary conditions are

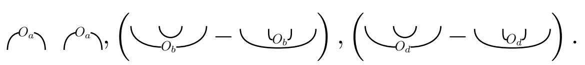
(45)

The boundary condition at either site b or d only accepts a $\perp$ spin above it (since the contraction with either $+$ or $-$ is zero). Hence if we average over the operator at site a, it has to be a $-$ spin so that domain wall and $\perp$ spin can be produced. We arrive at the boundary spin configurations in Supplementary Figure 6.

This is a special feature for the boundary condition at b (and also d). Let's label the four copies of the Hilbert space as 1, $\bar{1}$, 2, and $\bar{2}$. We make a permutation of those Hilbert space from $1\bar{1}2\bar{2}$ to $2\bar{2}1\bar{1}$. The tensor $u{\otimes}u^*{\otimes}u{\otimes}u^*$ (and consequently $+$, $-$ and $\perp$ states) and boundary condition at a remain invariant, but the boundary condition at b generates a $-$ sign. In a simpler example, this symmetry can be viewed the cyclic property of the trace

$$\mathrm{tr}([O_a(t), O_b]O_a(t)) = \mathrm{tr}(O_b[O_a(t), O_a(t)]) = 0. \tag{46}$$

In the off-diagonal OTOC, it precludes the situation in which b and d are separated by a surface with only $\pm$. For a proof, consider a configuration which surrounds site b and its neighboring unitaries with only $+, -$ spins and isolate it from d, such as the following

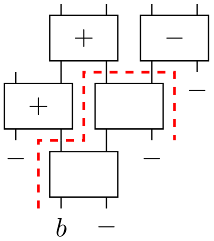

. (47)

Then the region enclosed by the red dash line has $\pm$ as its boundary conditions. We invoke the transformation from $1\bar{1}2\bar{2}$ to $2\bar{2}1\bar{1}$ *within the region enclosed by the red dash line.* Both the boundary $\pm$ spins and unitaries are invariant, however the boundary condition at b contributes $-$ sign. But since this is just the cyclic property of the trace, we conclude the weight of the enclosed region is zero, thus forbidding it. Therefore there should be a cluster of $\perp$ that connects b and d in the bulk.

If the distance x_{bd} between b and d is greater than $2t$, then there is no possibility for a $\perp$ cluster to connect them. We can then restrict x_{bd} to $2t$. A typical configuration is shown in Supplementary Figure 6. The two green curves represent the $\perp$ cluster that connects sites b and d. The two red lines represent the domain walls that seed the $\perp$ cluster. Compared to a local OTOC, the diagram suffers from two main suppressions. One is that the $-$ domain connecting the bottom boundary has size x_{bd}, while a local OTOC has at least $2v_Bt$. This brings in a factor of $q^{-(2v_Bt-x_{bd})}$. Another suppression comes from the $\perp$ cluster. Its relative weight with respect to an ordinary domain wall is $q^{-t_\perp}$ where $t_\perp$ is the persistent time of a cluster[17]. In this case, the suppression factor is $q^{-x_{bd}}$. The red and green two-segment domain wall is also not optimal, but we neglect this factor. Overall, the diagram in Supplementary Figure 6 can be a factor of $q^{-(2v_Bt-x_{bd}}q^{-x_{bd}} = q^{-2v_Bt}$ smaller than a local OTOC. Even if there can be $(2t)^2$ terms, the contribution is negligible than the v_Bt local OTOCs.

3. $a \neq c$, $b \neq d$

We have boundary conditions

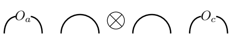
(48)

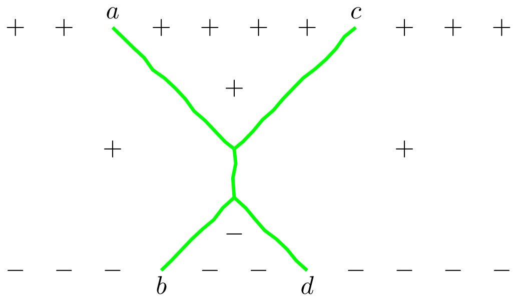

Supplementary Figure 7: Membrane configuration for $a \neq c$, $b \neq d$. There is a large $\perp$ cluster connecting a, b, c, d, resulting in an overall q^{-t} decay.

and

$$\left(\smile\smile - \smile_{O_b}\right) \otimes \left(\smile\smile - \smile_{O_d}\right). \qquad (49)$$

The analysis for site b and d is the same. They will be connected by a $\perp$ cluster in the middle. Sites a and c have to be directly connected to a $\perp$ spin. Since $\perp$ spin can be ended by a domain of the $+$ or $-$ spins, the $\perp$ clusters of site a and c have to join or meet the $\perp$ clusters of b and d. Such a large connected cluster gives a suppression of q^{-t}. Fixing the position of a, in order for all the $\perp$ clusters to meet, there are at most $(2t)^3$ terms. Hence the sum of all diagrams in Supplementary Figure 7 is at least order $t^3 q^{-t}$ smaller than the local OTOC, which is negligible in large t.

We conclude that when evolution is given by a local chaotic circuit, the asymptotic scaling of the global OTOC can be very well approximated by the sum of the local OTOCs. For local interactions, it is just the size of the light cone $2v_B t$.

B. Generalization to Long-range Interactions

We generalize the domain wall cost analysis to the off-diagonal terms when the interaction is long-ranged.

In the discussion of the local interaction, we see that when $b \neq d$, the $\perp$ cluster brings in a factor of $q^{-(v_B t - x_{bd})}$.

For case 2, this suppression factor can be generalized to $q^{-(2x_{\mathrm{LC}} - x_{bd})}$, where x_{LC} is the light cone size. We will sum over choices of b and d, both of which are within a region of $2x_{\mathrm{LC}}$ sites

$$\sum_{x_b=x_a-x_{\mathrm{LC}}}^{x_a+x_{\mathrm{LC}}} \sum_{x_d=x_a-x_{\mathrm{LC}}}^{x_a+x_{\mathrm{LC}}} q^{-(2x_{\mathrm{LC}}-|x_b-x_d|)} = q^{2x_{\mathrm{LC}}} \int_{-x_{\mathrm{LC}}}^{x_{\mathrm{LC}}} \int_{-x_{\mathrm{LC}}}^{x_{\mathrm{LC}}} dx_b dx_d q^{|x_b-x_d|} \sim \mathcal{O}(1/(\ln q)^2). \qquad (50)$$

Hence the contribution is of order 1, much smaller than x_{LC}, which is a lower bound for the sum of the local OTOCs. So case 2 is negligible.

For case 3, there is still a $\perp$ cluster connecting the four sites, although the spins can spread non-locally. But it is safe to say there is at least one $\perp$ spin at each time slice. Thus the suppressing factor of q^{-t} still works. There are at most x_{LC}^3 sites when fixing site a, so the sum is at the sale of $x_{\mathrm{LC}}^3 q^{-t}$. Since light cone spreads at most with a stretched exponential (for $\alpha > 0.5$), this sum for case 3 will be negligible for large t.

Therefore we still expect the sum of the local OTOCs to dominate.

[1] Y. Yen and A. Pines, Multiple-quantum NMR in solids, J. Chem. Phys. **78**, 3579 (1983).

[2] J. Baum, M. Munowitz, A. N. Garroway, and A. Pines, Multiple-quantum dynamics in solid state NMR, J. Chem. Phys. **83**, 2015 (1985).

[3] J. Baum and A. Pines, NMR studies of clustering in solids, J. Am. Chem. Soc. **108**, 7447 (1986).

[4] G. Cho and J. P. Yesinowski, H and 19F multiple-quantum NMR dynamics in quasi-one-dimensional spin clusters in apatites, J. Phys. Chem. **100**, 15716 (1996).

[5] I. Schnell and H. W. Spiess, High-resolution 1H NMR spectroscopy in the solid state: Very fast sample rotation and multiple-quantum coherences, J. Magn. Reson. **151**, 153 (2001).

[6] M. Munowitz and A. Pines, in *Advances in Chemical Physics*, edited by I. Prigogine and S. A. Rice (John Wiley & Sons, Inc., Hoboken, NJ, USA, 2007) pp. 1–152.

[7] C. M. Sánchez, H. M. Pastawski, and P. R. Levstein, Time evolution of multiple quantum coherences in NMR, Physica B **398**, 472 (2007).

[8] M. Munowitz, A. Pines, and M. Mehring, Multiple-quantum dynamics in NMR: A directed walk through liouville space, J. Chem. Phys. **86**, 3172 (1987).

[9] C. M. Sánchez, L. Buljubasich, H. M. Pastawski, and A. K. Chattah, Evolution of multiple quantum coherences with scaled dipolar Hamiltonian, J. Magn. Reson. **281**, 75 (2017).

[10] G. A. Álvarez, D. Suter, and R. Kaiser, Localization-delocalization transition in the dynamics of dipolar-coupled nuclear spins, Science **349**, 846 (2015).

[11] R. A. Jalabert and H. M. Pastawski, Environment-independent decoherence rate in classically chaotic systems, Phys. Rev. Lett. **86**, 2490 (2001).

[12] A. Khitrin, Growth of NMR multiple-quantum coherences in quasi-one-dimensional systems, Chem. Phys. Lett. **274**, 217 (1997).

[13] C. M. Sánchez, P. R. Levstein, L. Buljubasich, H. M. Pastawski, and A. K. Chattah, Quantum dynamics of excitations and decoherence in many-spin systems detected with loschmidt echoes: Its relation to their spread-

ing through the Hilbert space, Phil. Trans. R. Soc. A. **374**, 20150155 (2016).

[14] C. M. Sánchez, R. H. Acosta, P. R. Levstein, H. M. Pastawski, and A. K. Chattah, Clustering and decoherence of correlated spins under double quantum dynamics, Phys. Rev. A **90**, 042122 (2014).

[15] D. H. Levy and K. K. Gleason, Multiple quantum nuclear magnetic resonance as a probe for the dimensionality of hydrogen in polycrystalline powders and diamond films, J. Phys. Chem. **96**, 8125 (1992).

[16] F. D. Domínguez and G. A. Álvarez, Dynamics of quantum information scrambling under decoherence effects, arXiv:2107.03870 [cond-mat, physics:quant-ph] (2021).

[17] T. Zhou and A. Nahum, Entanglement membrane in chaotic many-body systems, Phys. Rev. X **10**, 031066 (2020).

[18] T. Zhou and A. Nahum, Emergent statistical mechanics of entanglement in random unitary circuits, Phys. Rev. B **99**, 174205 (2019).

[19] C. W. von Keyserlingk, T. Rakovszky, F. Pollmann, and S. L. Sondhi, Operator hydrodynamics, OTOCs, and entanglement growth in systems without conservation laws, Phys. Rev. X **8**, 021013 (2018).

[20] C. Jonay, D. A. Huse, and A. Nahum, Coarse-grained dynamics of operator and state entanglement, arXiv:1803.00089 [cond-mat, physics:hep-th, physics:nlin, physics:quant-ph] (2018).

[21] A. Nahum, J. Ruhman, S. Vijay, and J. Haah, Quantum entanglement growth under random unitary dynamics, Phys. Rev. X **7**, 031016 (2017).

[22] A. Nahum, S. Vijay, and J. Haah, Operator spreading in random unitary circuits, Phys. Rev. X **8**, 021014 (2018).
